# Neuropsychiatric and Cognitive Symptoms Across the Alzheimer Disease Clinical Spectrum: Cross-sectional and Longitudinal Associations

**DOI:** 10.1212/WNL.0000000000012598

**Published:** 2021-09-28

**Authors:** Willem S. Eikelboom, Esther van den Berg, Ellen H. Singleton, Sara J. Baart, Michiel Coesmans, Annebet E. Leeuwis, Charlotte E. Teunissen, Bart N.M. van Berckel, Yolande A.L. Pijnenburg, Philip Scheltens, Wiesje M. van der Flier, Rik Ossenkoppele, Janne M. Papma

**Affiliations:** From the Departments of Neurology (W.S.E., E.v.d.B., J.M.P.), Biostatistics (S.J.B.), and Psychiatry (M.C.), Erasmus MC, University Medical Center, Rotterdam; Department of Neurology, Alzheimer Center Amsterdam (E.H.S., A.E.L., Y.A.L.P., P.S., W.M.v.d.F., R.O.), Neurochemistry Laboratory, Department of Clinical Chemistry (C.E.T.), and Department of Radiology and Nuclear Medicine (B.N.M.v.B.), Amsterdam University Medical Centers, the Netherlands; and Clinical Memory Research Unit (R.O.), Lund University, Malmö, Sweden.

## Abstract

**Background and Objectives:**

To investigate the prevalence and trajectories of neuropsychiatric symptoms (NPS) in relation to cognitive functioning in a cohort of β-amyloid–positive (A+) individuals across the Alzheimer disease (AD) clinical spectrum.

**Methods:**

In this single-center observational study, we included all individuals who visited the Alzheimer Center Amsterdam and had a clinical diagnosis of subjective cognitive decline (SCD), mild cognitive impairment (MCI), or probable AD dementia and were A+. We measured NPS with the Neuropsychiatric Inventory (NPI), examining total scores and the presence of specific NPI domains. Cognition was assessed across 5 cognitive domains and with the Mini-Mental State Examination (MMSE). We examined trajectories including model-based trends for NPS and cognitive functioning over time. We used linear mixed models to relate baseline NPI scores to cognitive functioning at baseline (whole-sample) and longitudinal time points (subsample n = 520, mean 1.8 [SD 0.7] years follow-up).

**Results:**

We included 1,524 A+ individuals from the Amsterdam Dementia Cohort with A+ SCD (n = 113), A+ MCI (n = 321), or A+ AD dementia (n = 1,090). NPS were prevalent across all clinical AD stages (≥1 NPS 81.4% in SCD, 81.2% in MCI, 88.7% in dementia; ≥1 clinically relevant NPS 54.0% in SCD, 50.5% in MCI, 66.0% in dementia). Cognitive functioning showed a uniform gradual decline; while in contrast, large intraindividual heterogeneity of NPS was observed over time across all AD groups. At baseline, we found associations between NPS and cognition in dementia that were most pronounced for NPI total scores and MMSE (range β = −0.18 to −0.11, false discovery rate [FDR]–adjusted *p* < 0.05), while there were no cross-sectional relationships in SCD and MCI (range β = −0.32 to 0.36, all FDR-adjusted *p* > 0.05). There were no associations between baseline NPS and cognitive functioning over time in any clinical stage (range β = −0.13 to 0.44, all FDR-adjusted *p* > 0.05).

**Discussion:**

NPS and cognitive symptoms are both prevalent across the AD clinical spectrum, but show a different evolution during the course of the disease.

Alzheimer disease (AD) is characterized by a gradual decline in cognitive functions and activities of daily living.^1^ As neuropsychiatric symptoms (NPS) are present in the majority of patients with AD dementia,^2^ NPS are increasingly recognized as core clinical AD symptoms.^3^ Previous studies have associated the presence of NPS with an increased risk of progression to dementia and with worse cognitive performance and a faster cognitive decline in AD dementia.^4-6^ These studies have emphasized the clinical relevance of NPS in AD by highlighting its prognostic value.

Several other studies have not found an association between NPS and cognitive functioning in AD dementia.^7-9^ These discrepant results may have a number of causes, such as the fact that studies often used instruments that assess general cognitive functioning (i.e., Mini-Mental State Examination [MMSE]) and overall NPS burden (i.e., Neuropsychiatric Inventory [NPI] total score).^7,10^ Furthermore, prior studies have often included patients based on clinical diagnostic criteria of AD without biomarker evidence,^9,11^ thereby increasing the likelihood of including patients with non-AD primary etiologies.^12^

To address these challenges, the current study investigates (1) the prevalence and course of specific NPS and (2) associations between baseline NPS and performance on multiple cognitive domains at baseline and over time in a β-amyloid (Aβ)–positive (A+) sample ranging from normal cognition to dementia. This knowledge will provide a better understanding of the manifestation of NPS across the clinical stages of AD and its relationship with cognitive decline, which could aid patient management in clinical practice.

## Methods

### Participants

We included all patients who visited the Alzheimer Center Amsterdam between June 2002 and December 2017 and (1) had a clinical diagnosis of subjective cognitive decline (SCD), mild cognitive impairment (MCI), or probable AD dementia, (2) were A+, and (3) had NPI and neuropsychological assessment available at baseline. Individuals with possible AD dementia were excluded. All individuals underwent a standard diagnostic workup including medical history taking, neurologic examination, cognitive testing, lumbar puncture, and brain MRI.^13^ A subsample of the individuals underwent Aβ PET for research purposes (n = 450). Clinical diagnoses were established using conventional diagnostic criteria at multidisciplinary meetings. Individuals had to meet the clinical criteria of SCD,^14^ MCI,^15^ or probable AD dementia,^1^ in addition to Aβ positivity based on either CSF (i.e., Aβ_42_ < 550 pg/mL or tau/Aβ_42_ ratio > 0.52)^16^ or visual rating of an Aβ PET scan with the radiotracers ^18^F-florbetaben (n = 190), ^11^C-Pittsburgh compound B (n = 133), ^18^F-flutemetamol (n = 100), or ^18^F-florbetapir (n = 27).^17^ In case of Aβ PET/CSF discordance, Aβ status was determined based on the visual rating of Aβ PET. As all participants were Aβ-positive, SCD will be denoted as A+ SCD, MCI as A+ MCI, and AD dementia as A+ AD dementia.

### Neuropsychiatric Assessment

The NPI was used to assess NPS.^18^ This 12-item informant-based interview is a widely accepted measure of NPS in dementia.^3^ Each NPS domain is rated according to its severity (0–3) and frequency (0–4). We multiplied the severity and frequency scores for each domain to obtain an NPI domain score (0–12). The presence of specific NPS was defined as a severity × frequency score of ≥1 for each NPI domain. Clinically relevant NPS was defined as a severity × frequency score of ≥4 for each NPI domain. We summed the severity × frequency scores of all 12 domains to obtain the NPI total score (0–144). The presence of any NPS was defined as an NPI total score of ≥1. At baseline, scores were missing for the following NPI domains: n = 8 for eating behaviors, n = 8 for nighttime behaviors, n = 2 for aberrant motor behaviors, n = 1 for apathy, and n = 1 for agitation.

### Neuropsychological Assessment

We used the MMSE to assess global cognitive functioning. In addition, a standardized neuropsychological test battery was used to measure performance across 5 cognitive domains. We used immediate recall scores of the Visual Association Test part A and the immediate recall and delayed recall of the Rey Auditory Verbal Learning Test to measure memory. For attention, the Digit Span forward, Stroop Color and Word Test color and word conditions, and the Trail-Making Test part A were administered. Executive functioning was assessed using Digit Span backward, Stroop Color and Word Test color-word condition, Trail-Making Test part B, and the Frontal Assessment Battery. We used category fluency (animals) and the naming condition of the Visual Association Test to measure language. We measured visuospatial abilities using the number location, dot counting, and fragmented letters subtests of the Visual Object and Space Perception Battery.

Individuals who were not able to complete the Trail-Making Test or Stroop Color and Word Test due to cognitive difficulties were assigned the maximum score (i.e., higher scores reflect worse performance). We converted raw test scores into *Z* scores based on the mean and SD of an independent healthy reference group of 533 AD-biomarker negative individuals (mean [SD] age 59.7 [9.8], 54% female, mean [SD] MMSE score 28.9 [1.0]).^19^ The *Z* scores of the Trail-Making Test and Stroop test were inverted to ensure that lower scores indicated worse performance. Next, *Z* scores were combined into cognitive domain scores by averaging cognitive scores if at least 2 tests within that domain were available for that individual. At baseline, cognitive domain scores were missing for 7%–28%.

### Standard Protocol Approvals, Registrations, and Patient Consents

The Medical Ethics Review Committee of the Amsterdam University Medical Centers approved the study. Written informed consent was obtained from all participants.

### Statistical Analysis

We compared baseline clinical characteristics, NPS prevalence, and cognitive performance across the diagnostic groups using analysis of variance (with Tukey honestly significant difference post hoc test), Kruskal-Wallis tests, or χ^2^ tests where appropriate.

We aimed to statistically analyze trajectories of NPI scores but the assumption of normality was not met for the longitudinal NPI domain scores given the substantial proportion of zeros, which remained unchanged after deploying several transformations. Therefore, we plotted individual trajectories of NPS and cognitive functioning over time according to disease stage and added model-based trends with 95% confidence intervals (CIs) to the graphs for descriptive purposes. In addition, we investigated the extent to which NPS and cognitive functioning changed over time within individuals (intraindividual variance) and between individuals (interindividual variance). To quantify the variation within and between individuals, we conducted multilevel null models to obtain the percentage variance explained by intravariance and intervariance for neuropsychiatric measures and cognitive measures over time. For these analyses, the continuous severity × frequency scores (0–12) of specific NPI domains were used.

To study associations between baseline NPS and cognitive functioning at baseline and over time, we performed linear mixed models (LMMs) including random intercepts and fixed slopes that were corrected for age, sex, and education. Determinants included the NPI total score and the presence of specific NPI domains. Outcomes were performance on the MMSE and the 5 predefined cognitive domains. LMMs were run separately for the clinical stages at baseline (i.e., SCD, MCI, AD dementia). We tested nonlinear associations using LMMs with quadratic and cubic splines and selected linear LMM for all models based on the likelihood ratio χ^2^ test and Akaike information criterion. We checked assumptions by visual inspection of standardized residuals scatterplots and Q-Q plots. As normality of cognitive scores slightly deviated in language and visuospatial abilities most pronounced in A+ SCD, we conducted sensitivity analyses using a bootstrap procedure with 200 bootstrap samples to calculate CIs. This approach did not change the initial findings.

Level of significance was set at *p* < 0.05. The post hoc analyses on the NPS prevalence rates and the LMMs to study associations between NPS and cognitive performance were corrected for multiple testing using the Benjamini-Hochberg adjusted false discovery rate (FDR) of 0.05. Analyses were performed using SPSS version 26.0 and *R* version 4.0 (*lme4*, *splines*, *lmerTest*, *effectsize*, and *boot* packages).

### Data Availability

Data not provided in the article and additional information on methods and materials can be shared upon reasonable request.

## Results

### Participants

We included a total of 1,524 individuals of which 113 participants had a clinical diagnosis of A+ SCD, 321 participants with A+ MCI, and 1,090 participants with A+ AD dementia at baseline. Of the individuals with A+ AD dementia at baseline, the majority had mild dementia (87.2%, clinical dementia rating [CDR] score ≤1), while 12.4% had moderate dementia (CDR = 2), and 0.5% had severe dementia (CDR = 3). A subsample of the participants had follow-up assessments available: n = 53 (46.9%) with A+ SCD at baseline, n = 142 (44.2%) with A+ MCI at baseline, and n = 326 (29.9%) with A+ AD dementia at baseline. We found no differences in demographic and clinical characteristics between individuals with and without follow-up assessments for A+ SCD and A+ MCI. In A+ AD dementia, we did find lower cognitive functioning and higher NPS burden in individuals without follow-up assessment compared to individuals with follow-up assessment (eTable 1, doi.org/10.5061/dryad.hqbzkh1g2). We conducted longitudinal analyses in patients who had follow-up assessments available limited up to 3 years after baseline assessment, because <10% of the 1,524 participants had more than 3 years of follow-up assessments available. Including these assessments may have resulted in underestimation of disease progression due to selective dropout.^20^ For those with follow-up assessment available, mean follow-up duration was 1.7 years (SD 0.8) for A+ SCD, 1.9 years (SD 0.7) for A+ MCI, and 1.7 years (SD 0.7) for A+ AD dementia.

Baseline demographic and clinical characteristics of our sample of A+ individuals are shown in Table 1. Participants with A+ MCI were older than individuals with A+ AD dementia (*p* < 0.001) or A+ SCD (*p* < 0.05). Participants with A+ AD dementia had lower levels of education compared to those without dementia (*p* < 0.001). The proportion of female patients was higher in A+ AD dementia than A+ MCI (*p* < 0.001). Of the individuals without dementia at baseline who had follow-up assessment available, 24.5% (n = 13) of the individuals with A+ SCD progressed to MCI or dementia, and 43.0% (n = 61) of the participants with A+ MCI progressed to dementia. As expected, baseline MMSE and baseline cognitive domain scores differed according to clinical AD stage (*p* < 0.001; Table 1).

**Table 1:**
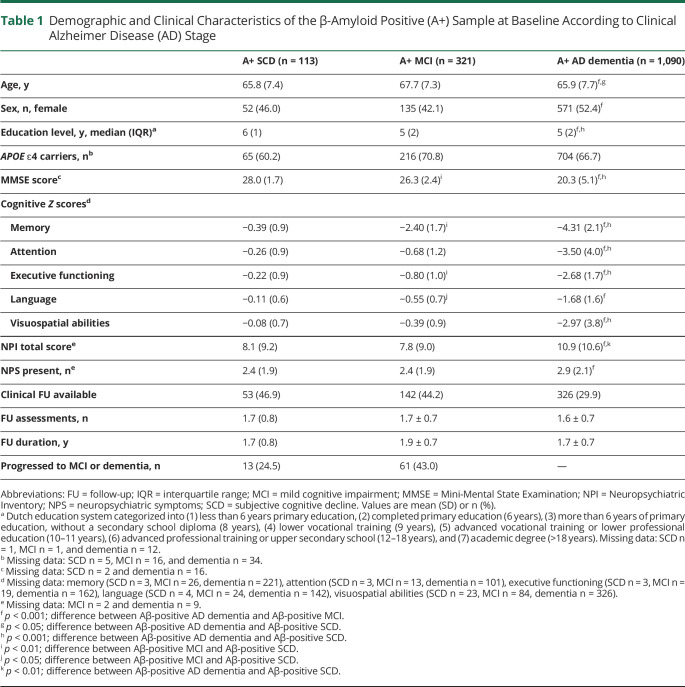
Demographic and Clinical Characteristics of the β-Amyloid Positive (A+) Sample at Baseline According to Clinical Alzheimer Disease (AD) Stage

### Prevalence of NPS at Baseline Across Clinical Stages

**Figure 1:**
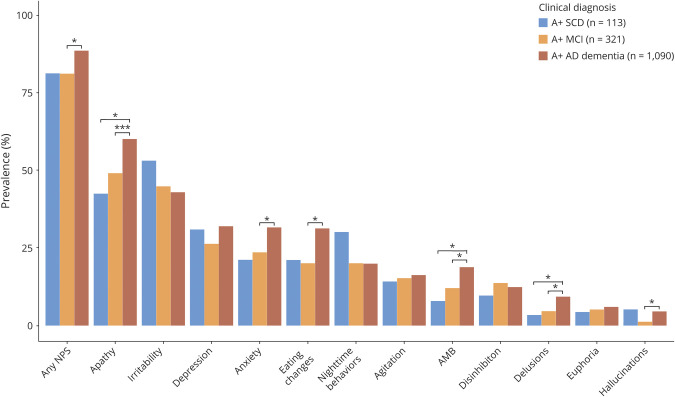
Prevalence of Neuropsychiatric Symptoms (NPS) Across a β-Amyloid–Positive (A+) Sample According to Clinical Alzheimer Disease (AD) Stage *p < 0.05 after correcting for false discovery rate. **p < 0.01 after correcting for false discovery rate. ***p < 0.001 after correcting for false discovery rate. AMB = aberrant motor behaviors; MCI = mild cognitive impairment; SCD = subjective cognitive decline.

NPS were prevalent across all AD stages with at least one NPS present in 81.4% of the individuals with A+ SCD (54.0% rated as clinically relevant), 81.2% of the individuals with A+ MCI (50.5% rated as clinically relevant), and 88.7% of the individuals with A+ AD dementia (66.0% rated as clinically relevant). The NPI total score was higher for A+ AD dementia compared to A+ SCD (*p* < 0.01) and A+ MCI (*p* < 0.001), while we found no difference in NPI total score between A+ SCD and A+ MCI (*p* = 0.97; Table 1). The number of NPS present at baseline was higher for A+ AD dementia compared to A+ MCI (*p* < 0.001), with no difference between A+ SCD and the other clinical stages (all *p* > 0.05, Table 1). The prevalence rates of the specific NPI domains across the clinical AD stages are presented in Figure 1. The 3 most prevalent NPS were similar for all clinical stages and included apathy, irritability, and depression. The prevalence was higher at the more advanced clinical stage for the majority of NPI domains, especially for apathy, anxiety, eating behaviors, aberrant motor behaviors, and delusions. However, irritability, depression, nighttime behaviors, and hallucinations were more common in A+ SCD compared to A+ MCI or A+ AD dementia.

The NPI severity scores and frequency scores showed a similar pattern as the NPI prevalence rates, i.e., the highest severity and frequency scores were seen in A+ AD dementia with little differences between A+ SCD and A+ MCI (eTables 2 and 3, doi.org/10.5061/dryad.hqbzkh1g2). Furthermore, the distribution of clinically relevant NPS (NPI domain scores ≥4) showed a similar pattern compared to the distribution of the presence of NPS (eTable 2, doi.org/10.5061/dryad.hqbzkh1g2).

### Progression of NPS and Cognition Over Time Across Clinical Stages

**Figure 2:**
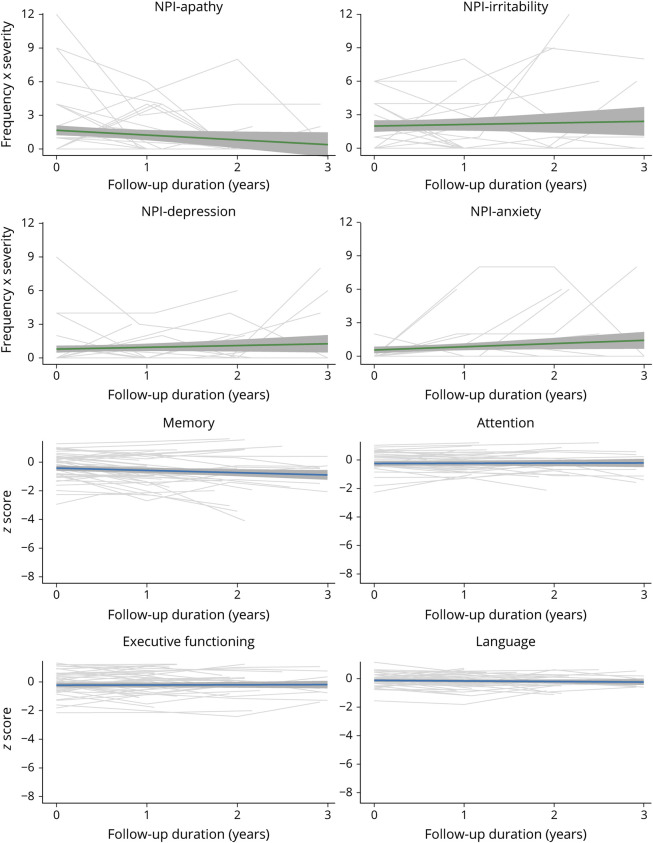
Longitudinal Neuropsychiatric Inventory (NPI) Domain Scores and Cognitive Functioning for Individuals With β-Amyloid–Positive Subjective Cognitive Decline at Baseline Individual trajectories are depicted with model based trends with 95% confidence intervals. Data show the 4 most prevalent neuropsychiatric symptoms at baseline and cognitive domains with most data available. See eFigure 1 (doi.org/10.5061/dryad.hqbzkh1g2) for all data.

**Figure 3:**
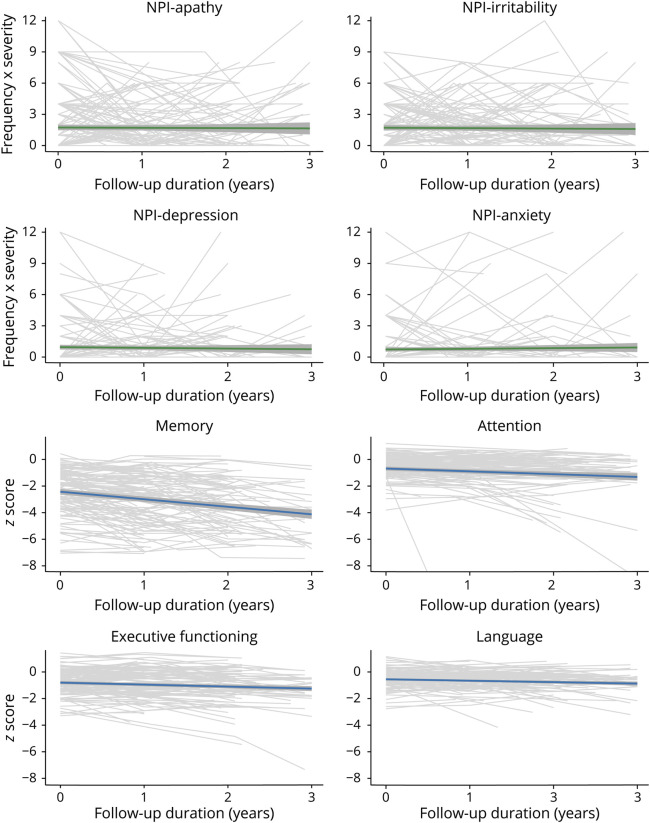
Longitudinal Neuropsychiatric Inventory (NPI) Domain Scores and Cognitive Functioning for Patients With β-Amyloid–Positive Mild Cognitive Impairment at Baseline Individual trajectories are depicted with model based trends with 95% confidence intervals. Data show the 4 most prevalent neuropsychiatric symptoms at baseline and cognitive domains with most data available. See eFigure 2 (doi.org/10.5061/dryad.hqbzkh1g2) for all data.

**Figure 4:**
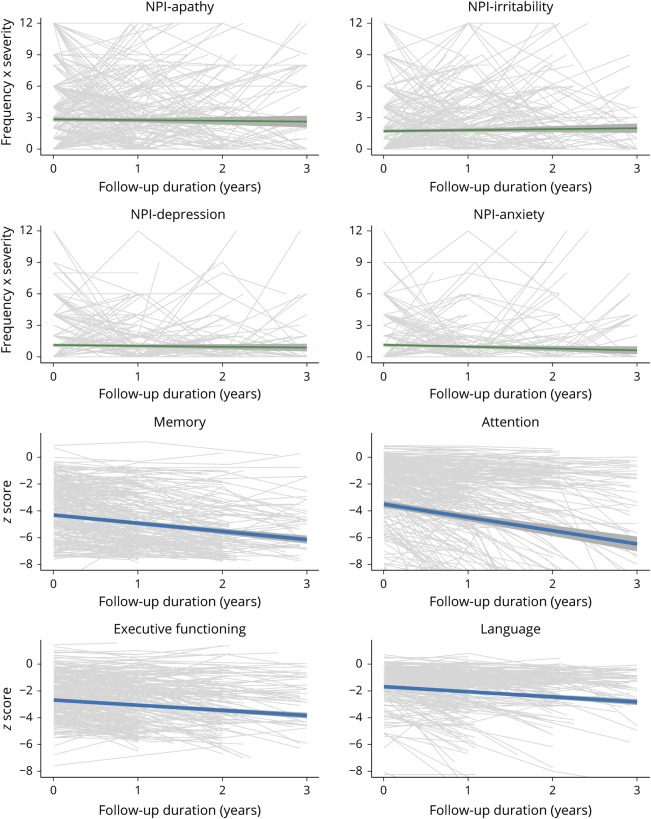
Longitudinal Neuropsychiatric Inventory (NPI) Domain Scores and Cognitive Functioning for Patients With β-Amyloid–Positive Alzheimer Disease Dementia at Baseline Individual trajectories are depicted with model based trends with 95% confidence intervals. Data show the 4 most prevalent neuropsychiatric symptoms at baseline and cognitive domains with most data available. See eFigure 3 (doi.org/10.5061/dryad.hqbzkh1g2) for all data.

We plotted trajectories of specific NPI domains and performance on specific cognitive domains over time for patients across the different clinical AD stages. In participants with A+ SCD at baseline, the trends of specific NPI domain scores remained stable over time with a decline in apathy and a subtle increase for depression, anxiety, and agitation (Figure 2, eFigure 1, doi.org/10.5061/dryad.hqbzkh1g2). Cognitive scores remained relatively stable over time for A+ SCD. In participants with A+ MCI at baseline, we observed a relatively stable trend of specific NPI domains over time, whereas a decline was observed in all cognitive domains (Figure 3, eFigure 2, doi.org/10.5061/dryad.hqbzkh1g2). In participants with A+ AD dementia, few changes were found in trends of specific NPI domains over time, with modest increases in irritability, aberrant motor behaviors, and nighttime behaviors and decrease in depression and anxiety (Figure 4, eFigure 3, doi.org/10.5061/dryad.hqbzkh1g2). Substantial decline was observed in all cognitive domains.

When looking at the trajectories of specific NPS and cognitive scores over time, we observed large variability in the course of specific NPS within and between individuals across all clinical stages (Figures 2–4, eFigures 1–3, doi.org/10.5061/dryad.hqbzkh1g2). To further quantify this intraindividual variability and interindividual variability, we performed multilevel null models for each measure according to clinical stage at baseline (eTable 4, doi.org/10.5061/dryad.hqbzkh1g2). Across all clinical AD stages, the intraindividual variance of NPS measures was higher (all mean % explained >70%) compared to cognitive measures (all mean % explained <45%). Hence, we observed larger changes on NPS measures over time within individuals than between individuals, while the opposite was the case for cognitive measures.

### Cross-sectional Associations Between NPS and Cognitive Functioning at Baseline

Age-, sex-, and education-corrected LMM in A+ AD dementia showed that higher baseline NPI total scores were associated with lower baseline MMSE scores (β = −0.08, 95% CI [−0.14, −0.02], FDR-adjusted *p* < 0.001) and lower performance on visuospatial abilities (β = −0.11, 95% CI [−0.18, −0.04], FDR-adjusted *p* < 0.05). Baseline NPI total scores were not related to cognitive functioning at baseline in A+ SCD and A+ MCI (all FDR-adjusted *p* > 0.05, eTable 5, doi.org/10.5061/dryad.hqbzkh1g2).

Next, age-, sex-, and education-corrected LMM assessing the associations between the presence of specific NPS and baseline cognitive performance showed that the presence of aberrant motor behaviors (β = −0.28 [−0.43, −0.13], FDR-adjusted *p* < 0.05), agitation (β = −0.27 [−0.43, −0.11], FDR-adjusted *p* < 0.05), euphoria (β = −0.27 [−0.41, −0.12], FDR-adjusted *p* < 0.05), and apathy (β = −0.18 [−0.30, −0.06], FDR-adjusted *p* < 0.05) were associated with worse MMSE scores in A+ AD dementia. The presence of nighttime behaviors was related to worse performance in language in AD dementia (β = −0.29 [−0.44, −0.13], FDR-adjusted *p* < 0.05). In A+ MCI, the presence of hallucinations was associated with worse performance in attention (β = −1.73 [−2.68, −0.79], FDR-adjusted *p* < 0.05). We found no associations between the presence of specific NPS and cognitive functioning at baseline in A+ SCD (all FDR-adjusted *p* > 0.05, eTable 5, doi.org/10.5061/dryad.hqbzkh1g2).

Repeating age-, sex-, and education-corrected LMM for clinically relevant NPS (NPI domain score ≥ 4) yielded highly similar results (eTable 6, doi.org/10.5061/dryad.hqbzkh1g2).

### Associations Between Baseline NPS and Cognitive Functioning Over Time

Using LMMs adjusted for age, sex, and education, baseline NPI total scores were not associated with changes in MMSE scores or cognitive domains over time in our cohort of A+ individuals ranging from SCD to AD dementia (all FDR-adjusted *p* > 0.05, eTable 7, doi.org/10.5061/dryad.hqbzkh1g2). With regard to specific NPS, baseline irritability was associated with less steep memory decline over time in individuals with A+ SCD at baseline (β = 0.44 [0.26, 0.61], FDR-adjusted *p* < 0.001). None of the baseline NPI domain scores was associated with cognitive functioning over time in A+ MCI and A+ AD dementia (all FDR-adjusted *p* > 0.05, eTable 7, doi.org/10.5061/dryad.hqbzkh1g2).

Repeating these age-, sex-, and education-corrected LMM with the presence of clinically relevant NPS (NPI domain score ≥ 4) did not change our findings (eTable 8, doi.org/10.5061/dryad.hqbzkh1g2).

## Discussion

The main findings in this A+ sample are (1) high prevalence rates of NPS across all clinical AD stages, (2) a substantial heterogeneity in trajectories of NPS over time, (3) cross-sectional associations between the presence and severity of NPS and worse cognitive functioning in dementia, and (4) absence of clear associations between baseline NPS and performance on specific cognitive domains over time for all clinical AD stages.

NPS were prevalent across the entire clinical AD spectrum and showed little relation with clinical severity. Almost 90% of the patients with A+ AD dementia showed at least one NPS, which is in line with previous studies.^2,21^ Furthermore, over 80% of the individuals in the predementia AD stages exhibited at least one NPS, which is remarkably higher compared to prior studies.^6,7^ Although NPS prevalence and severity was higher in AD dementia compared to predementia AD stages, our findings suggest that NPS may precede cognitive impairment during the clinical course of AD. These findings support the construct of mild behavioral impairment (MBI) by recognizing that NPS can be an early manifestation of dementia.^22^ We were not able to establish the prevalence of MBI in our study as we had no information on the duration and degree of impairment of the NPS in the predementia stages. We did not find many differences in NPS prevalence and severity between individuals with A+ SCD and individuals with A+ MCI, while some specific NPS were even more prevalent in A+ SCD compared to A+ MCI. NPS that were common in the A+ SCD stage included affective symptoms, irritability, and nighttime behaviors and might be a psychological response to the initial cognitive decline experienced and might be a reason to visit the memory clinic.^23^ Prior studies have indeed shown higher NPS prevalence rates in predementia memory clinic cohorts compared to population-based studies.^2,23,24^ Moreover, the high NPS prevalence observed across all clinical stages in our cohort may also be influenced by the fact that these individuals visited a tertiary academic memory clinic with an overrepresentation of early-onset and atypical AD.

Our results indicate a different evolution over time for NPS compared to cognitive symptoms across the AD clinical spectrum. As expected, cognitive functioning showed a gradual decline that was most pronounced in the dementia stage.^25^ In contrast, the course of NPS showed a less coherent pattern with a relatively stable trends across all clinical AD stages, which is in line with prior research.^26,27^ Moreover, we found substantial heterogeneity within individuals in their course of NPS compared to the intraindividual variation of the course of cognitive functioning over time. Although previous studies have also suggested large variability in NPS prevalence and evolution between and within patients,^28-30^ the fluctuations in NPS observed in our study may also be due to methodologic aspects of NPS measurements. For example, while cognitive functioning was assessed by an extensive neuropsychological assessment covering 5 cognitive domains with at least 2 cognitive tests for each domain, NPS were measured using a single caregiver rating scale that can be affected by caregiver distress and recall bias.^3^ To obtain better insight in fluctuations in NPS in AD, future studies could assess NPS on short time intervals using a combination of informant-based scales, clinician-rating instruments, and self-report measures, e.g., by using an Ecological Momentary Assessments approach in which existing NPS scales are adjusted for daily assessments.^31^

At baseline, we found associations between the presence and severity of NPS and lower cognitive performance in patients with AD dementia that were most evident when looking at NPI total scores and MMSE performance. We found little evidence for a cross-sectional relationship between NPS and cognitive functioning in the predementia AD stages. Cross-sectional relationships between NPS and cognitive deficits as measured with the MMSE have previously been reported in AD dementia,^32,33^ and associations between NPS and performance on specific cognitive domains have also rarely been found in AD dementia.^5,8,34,35^ These differences in cross-sectional associations between clinical AD stages found in this study may be explained by the larger degree of cognitive variability among individuals with AD dementia compared to predementia stages. We found no clear associations between baseline NPS and cognitive functioning over time across clinical stages. Although prior studies have yielded similar results in different cohorts of patients with AD dementia,^9,34,35^ our findings are in contrast to several other studies that have related the presence of NPS with accelerated cognitive decline in individuals with normal cognition,^11,36^ MCI,^10^ and AD dementia.^4,37^ Previous studies have suggested that NPS are an integral part of AD and that the presence of NPS may be suggestive of underlying AD pathology.^38^ The studies described above that have previously examined the relationship between NPS and cognitive functioning have primarily included samples without AD biomarker evidence. As a consequence, the presence of NPS in these samples may be suggestive of underlying AD pathology and has therefore been associated with cognitive decline. However, we already substantially increased the likelihood of underlying AD pathology as all individuals in our sample were A+. Consequently, the presence of NPS may have less predictive value in this sample of A+ individuals.

Our findings provide useful information for the management of care for patients with AD. While one can expect a gradual decline of cognitive functioning over time, it appears difficult to predict the progression of NPS given the large differences between and within individuals despite group trajectories showing generally little change over time. These findings emphasize a patient-centered approach in the assessment and management of NPS across all clinical AD stages. Moreover, more future studies are needed that focus on identifying subgroups of individuals at risk for developing NPS.

Cognitive symptoms have been related to pathophysiologic and neurodegenerative processes in AD, with generally weaker associations with Aβ as compared to brain atrophy and tau pathology.^39^ Several theories have been proposed to explain the manifestation of NPS in AD.^3,40^ While the symptom hypothesis states that NPS result from AD-related neuropathology that also contributes to cognitive impairment in AD, the risk factor hypothesis suggests that NPS arise from concurrent non-AD pathology, e.g., vascular depression.^40,41^ Recent studies have reported inconsistent associations between NPS and AD pathology,^42-44^ while providing some evidence for associations with non-AD biomarkers.^40,45,46^ Our findings suggested substantial fluctuations over time with no coherent pattern of decline or increase in NPS as the disease progresses, leaving room open for other factors affecting NPS in AD. In addition to neurobiological causes, a variety of psychosocial factors have been proposed to play a role in the emergence and worsening of NPS in AD, including unmet needs, stress among caregivers, and environmental triggers.^47^ Our findings show substantial fluctuations in NPS over time and no clear associations with cognitive symptoms, suggesting that the symptom hypothesis alone cannot explain the emergence of NPS in AD.

Strengths of this study include the large well-defined sample of individuals who were all A+ and underwent an extensive neuropsychological battery used to assess cognitive functioning. However, this study also has some limitations. First, although we took a unilateral perspective when examining the relationship between NPS and cognitive functioning, we acknowledge that cognitive impairments can also contribute to the presence and worsening of NPS in AD.^48^ Second, we examined a relatively young cohort of participants (mean [SD] age 66.3 [7.7]) who visited a tertiary memory clinic and may be characterized by a relative absence of age-related comorbidities. This may limit the generalizability of our findings to cohorts with older individuals with AD. Furthermore, the number of individuals with A+ SCD or A+ MCI at baseline with follow-up assessments was low, resulting in low power and increasing potential risk of bias for these analyses. Analyses of potential bias in loss to follow-up of individuals showed little bias in A+ SCD and A+ MCI, but did show that individuals without follow-up assessments had more severe A+ AD dementia and greater NPS burden. Future studies with larger sample sizes including individuals with severe AD dementia are therefore needed. In addition, we did not have information on the use of psychotropic drugs, cholinesterase inhibitors, and memantine. This is an important limitation as these medications may affect the prevalence and evolution of both cognitive and neuropsychiatric symptoms. Finally, we were not able to formally test NPS trajectories using LMM, as assumptions of normality and linearity were not met. This was caused by a substantial proportion of zero scores on the NPI, as well as the way NPI domain scores are calculated, i.e., by multiplying the severity score of 0–3 by the frequency score of 0–4 so that the values 5, 7, 10, and 11 cannot be observed.^49,50^ Using symptom-specific instruments such as the Apathy Evaluation Scale (score range 18–71), Cohen-Mansfield Agitation Inventory (score range 29–203), and Geriatric Depression Scale (score range 0–30) may not only help to fully characterize specific NPS, but also enables the use of statistical modeling due to a larger variation in potential scores compared to the NPI.

To conclude, NPS were prevalent in a well-defined Aβ-positive sample ranging from normal cognition to dementia. We found little association between NPS and cognitive symptoms at baseline and over time across the AD clinical spectrum. These findings suggest that NPS and cognitive symptoms are independent manifestations of AD that show a different evolution over the course of the disease.

## Publication History

Previously published in medRxiv (doi.org/10.1101/2021.02.18.21251064).

Received by *Neurology* February 24, 2021. Accepted in final form June 29, 2021.

## Glossary

A+: β-amyloid–positive
Aβ: β-amyloid
AD: Alzheimer disease
CDR: clinical dementia rating
CI: confidence interval
FDR: false discovery rate
LMM: linear mixed model
MBI: mild behavioral impairment
MCI: mild cognitive impairment
MMSE: Mini-Mental State Examination
NPI: Neuropsychiatric Inventory
NPS: neuropsychiatric symptoms
SCD: subjective cognitive decline
